# A Subgroup of Patients With Hospital-acquired Pneumonia Do Not Require Broad-spectrum Gram-negative Antimicrobial Coverage

**DOI:** 10.1093/cid/ciaa391

**Published:** 2020-04-08

**Authors:** Clark D Russell, Ed Whittaker, Dominic P Dee, Eilidh Farquhar, Alba Saenz de Villaverde, Morgan H Evans, Ian F Laurenson, Claire L Mackintosh, Muge Cevik

**Affiliations:** 1 University of Edinburgh Centre for Inflammation Research, Queen’s Medical Research Institute, Edinburgh BioQuarter, Edinburgh, United Kingdom; 2 National Health Service Lothian Infection Service, Regional Infectious Diseases Unit, Western General Hospital, Edinburgh, United Kingdom; 3 National Health Service Lothian Infection Service, Clinical Microbiology, Royal Infirmary of Edinburgh, Edinburgh, United Kingdom; 4 Infection and Global Health Research, School of Medicine, University of St Andrews, Fife, United Kingdom

**Keywords:** hospital-acquired pneumonia, Gram-negative bacteria, doxycycline, gentamicin, antimicrobial stewardship

## Abstract

Among 200 patients developing hospital-acquired pneumonia (HAP) outside the intensive care unit, 61% were treated empirically without broad-spectrum Gram-negative coverage, with clinical cure in 69.7%. Lower disease severity markers (systemic inflammatory response syndrome, hypoxia, tachypnoea, neutrophilia) and the absence of diabetes mellitus and prior doxycycline treatment (but not the time to HAP onset) identified patients not requiring broad-spectrum Gram-negative coverage.

Hospital-acquired pneumonia (HAP) occurring outside the intensive care unit (ICU) is a relatively understudied nosocomial infection (non-ICU HAP). Gram-negative bacilli (GNB) such as Enterobacteriaceae and *Pseudomonas aeruginosa* are considered canonical HAP pathogens and international guidelines recommend empiric broad-spectrum Gram-negative antimicrobial coverage [1, 2]. However, much of the literature actually describes ventilator-associated pneumonia or ICU HAP. Case ascertainment bias exists in other studies, reporting only on patients able to expectorate sputum or with positive sputum cultures, whereas real-life data demonstrate that sputum samples are infrequently available and often culture-negative [3–6]. Therefore, our understanding of the microbial aetiology of non-ICU HAP is incomplete, and empiric broad-spectrum Gram-negative coverage may not be mandated in all cases. In the United Kingdom, doxycycline is widely recommended for the empiric treatment of low-severity HAP, but we are aware of no clinical data supporting this practice [7]. Doxycycline lacks activity against Enterobacteriaceae, *P. aeruginosa*, and *Acinetobacter baumanii*; thus, its usage provides an opportunity to address the unanswered question of the requirement for broad-spectrum Gram-negative coverage in non-ICU HAP. The aims of this study were to (1) identify and characterize a representative cohort of patients with non-ICU HAP; and (2) report treatment outcomes without broad-spectrum Gram-negative coverage.

## METHODS

### Case Ascertainment

An electronic search identified all inpatient ward chest X-rays (CXR) performed in 2 tertiary care hospitals in Edinburgh, United Kingdom, over 13 months (June 2018–July 2019) where the request or report included the terms “consolidation” or “pneumonia” (n = 4250). These reports were reviewed to identify those cases with radiological evidence of infective consolidation (n = 728). The electronic patient records for these patients were reviewed to determine whether the case definition was met. Non-ICU HAP was defined as new/progressive CXR consolidation occurring (1) ≥48 hours after hospital admission; (2) in a non-intubated adult; (3) in a non-ICU ward (and ≥48 hours since ICU discharge if relevant), (with ICU defined as a unit capable of providing mechanical ventilation); (4) with documentation of consistent symptoms (cough, sputum, pleuritic chest pain, dyspnoea) or chest auscultation findings (crackles, reduced air entry, bronchial breathing); and (5) a clinical diagnosis of pneumonia. Patients were excluded if pneumonia occurred following a frank aspiration event or if a clinical diagnosis of aspiration pneumonia was made.

### Data Collection

Relevant clinical, laboratory, and microbiological details for the first HAP episode were recorded from the electronic patient records. Clinical cure was defined as the treatment of HAP without a requirement for antimicrobial escalation and without mortality attributable to HAP (treatment failure refers to the occurrence of 1 or both outcomes). Mortality was considered attributable to HAP if pneumonia was recorded on the death certificate.

### Statistical Analysis

Continuous variables were compared using an unpaired *t*-test if normally distributed or a Mann Whitney test if not. Categorical variables were compared using Fisher’s exact test. Following a receiver operator characteristic analysis, Youden’s J statistic was calculated to determine cut-offs for the association between continuous variables and outcomes. Variables identified by univariate analysis were included in multiple logistic regression after assessing multicollinearity. We reported 2-tailed *P*-values, and a *P* < .05 was considered statistically significant. The statistical analysis was performed using Prism, version 8.0 (GraphPad Software Inc, San Diego, CA).

### Ethical Approval

A favorable ethical opinion was provided by the West of Scotland Research Ethics Service (19/WS/0152) and Caldicott approval was provided by National Health Service Lothian Research and Development (2019/0242).

## RESULTS

### Characteristics of Patients Developing Non–Intensive Care Unit Hospital-acquired Pneumonia

We identified 200 patients with non-ICU HAP (Table 1). Patients had a median age of 77 years and 43% were aged ≥80 years. A median of 2 (interquartile range [IQR], 1–3) medical comorbidities were present per patient, most commonly chronic lung disease (78%). During the preceding year, 57.5% of patients had been hospitalized (median, 1 prior admission; IQR, 0–2). Common admission events preceding HAP included receipt of antimicrobials (53.5%) and surgery (34%). Methicillin-resistant *Staphylococcus aureus* carriage was identified in 4/132 screens performed. 11.5% of patients were colonized with other antimicrobial-resistant organisms (vancomycin-resistant enterococci, n = 7; multidrug-resistant GNB in urine, n = 15; carbapenemase-producing organism, n = 1).

**Table 1.** Clinical, Laboratory, and Outcome Data

Variable	n (%)
Patient characteristics	
Age, median (IQR) years	77 (62–87)
Male	110 (55)
Comorbidities	
Chronic lung disease	78 (39)
Ischaemic heart disease or heart failure	74 (37)
Cerebrovascular disease	57 (28.5)
Diabetes mellitus	49 (24.5)
Chronic kidney disease	46 (23)^a^
Solid cancer	45 (22.5)
Immunosuppression^b^	19 (9.5)
Liver cirrhosis	15 (7.5)
Hematological malignancy	10 (5)
Current smoker	35 (17.5)
Time to onset of HAP, median (IQR) days	9 (5–21)
Admitting speciality	
Medicine	119 (59.5)
Surgery	81 (40.5)
Admission events preceding HAP	
Prior antimicrobials	107 (53.5)
Surgery	68 (34)
Other infection	66 (33)
Endotracheal intubation^c^	50 (25)
Bone fracture	38 (19)
ICU admission	30 (15)
Community-acquired LRTI or pneumonia	42 (21)
HAP episode^d^	
Physiological parameters	
Sepsis (qSOFA ≥ 2)	19 (9.5)
SIRS (SIRS ≥ 2)	102 (51)
New/increased supplemental O_2_ requirement	129 (64.5)
Altered mentation	41 (20.5)
Temperature ≥38.0°C or <36°C	80 (40)
Heart rate, mean (SD) beats/minute	95 (±22)
Systolic blood pressure, median (IQR) mmHg	119 (107–132)
Respiratory rate, median (IQR) breaths/minute	20 (17–24)
Intravenous fluid resuscitation	38 (19)
ICU admission	9 (4.5)
Laboratory parameters	
New acute kidney injury^e^	24 (12)
Total white cell count, ×10^9^ L^−1^, median (IQR)	10.6 (7.7–14.7)
Neutrophil count, ×10^9^ L^−1^, median (IQR)	8.4 (5.7–11.8)
Lymphocyte count, ×10^9^ L^−1^, median (IQR)	1.0 (0.7–1.6)
C-reactive protein, mg L^−1^, median (IQR)	94 (38–190)
Outcomes	
Clinical cure	140 (70)
HAP antimicrobial escalation	36 (18)
Mortality attributable to HAP	32 (16)
Further episode of HAP	33 (16.5)

Data for included patients with non-ICU hospital-acquired pneumonia (n = 200).

Abbreviations: HAP, hospital-acquired pneumonia; HIV, human immunodeficiency virus; ICU, intensive care unit; IQR, interquartile range; LRTI, lower respiratory tract infection; SD, standard deviation; SIRS, systemic inflammatory response syndrome; qSOFA, quick Sequential (sepsis-related) Organ Failure Assessment score.

^a^Requiring haemodialysis in 5 cases.

^b^Therapeutic immunosuppression (n = 13), antineoplastic chemotherapy (n = 3), living with HIV (n = 2), and immunoglobin G sub-class 2 and 3 deficiency (n = 1).

^c^For general anaesthesia or mechanical ventilation.

^d^Physiological and laboratory parameters obtained within 24 hours of HAP diagnosis were recorded.

^e^Increase in creatinine of ≥26.5 μmol/L from last measurement.

### Clinical and Microbiological Features of Non-ICU HAP

HAP was diagnosed a median of 9 days after admission. Sepsis (quick Sequential [sepsis-related] Organ Failure Assessment score [qSOFA] ≥ 2 [8]) was present in 9.5% of patients and systemic inflammatory response syndrome (SIRS, ≥2 criteria) was present in 51%. Hypoxia necessitating new or increased supplemental oxygen was common (64.5%) but extra-pulmonary organ dysfunction was less common, indicated by a low requirement for intravenous fluid resuscitation (19%) and low incidences of altered mentation (20.5%) and acute kidney injury (12%). The median white cell count was in the normal range (10.6 × 10^9^/L), but the median C-reactive protein was elevated (94 mg/L) and lymphopenia was common (70%). Microbiological evaluation consisted of a sputum sample in 18.5% of patients, a respiratory pathogen polymerase chain reaction throat swab in 27%, and blood cultures in 47.5%. It was not known whether samples were obtained prior to antimicrobials. Pathogenic bacteria were identified in 19/37 sputum samples and respiratory viruses were identified in 18/54 swabs (Supplementary Table 1). There was 1 patient with *Escherichia coli* bacteremia (1/95 blood cultures). There were 12 patients who had microbiological evidence of HAP caused by Enterobacteriaceae or *P. aeruginosa*. This was associated with the number of comorbidities (median, 3 vs 2; *P* = .035) and specifically with chronic obstructive pulmonary disease (58.3% vs 23.4%; *P* = .013), but not with time to HAP onset, prior hospitalization, or prior antimicrobials.

### Management and Outcomes

The most common first-line empiric antimicrobial used was doxycycline (59.5%), followed by amoxicillin and gentamicin (14%), vancomycin and gentamicin (6.5%), and then piperacillin-tazobactam (5.5%; Supplementary Table 2). Antimicrobial escalation was required in 18% of cases, most commonly from doxycycline to a regimen including broader Gram-negative coverage (29/36 instances). The median total duration of antimicrobials was 7 days (IQR, 5–7).

Clinical cure was achieved in 70% of patients. Mortality attributable to HAP occurred in 16% of patients. Logistic regression identified a new/increased supplemental oxygen requirement (odds ratio [OR], 5.5; 95% confidence interval [CI], 2.4–13.9; *P* = .0002) and urea >5.5 mmol/L (OR 4.6; 95% CI 1.9–12.9; *P* = .002) as being associated with treatment failure. Undergoing surgery prior to developing HAP was associated with a reduced likelihood of treatment failure (OR, 0.4; 95% CI, .2–1.0; *P* = .04). A further episode of HAP during the same admission occurred in 16.5% of patients, and there were 2 cases of *Clostridioides difficile* infection following HAP treatment. A HAP diagnosis was recorded on discharge documentation for coding in 42.5% of cases.

### Patients Treated Without Empiric Broad-spectrum Gram-negative Coverage

Overall, 61% of patients were treated without empiric broad-spectrum Gram-negative coverage, with clinical cures in 69.7%. These patients had lower SIRS scores when compared to patients treated empirically with such coverage (median, 1 vs 2, respectively; *P* < .0001), consistent with institutional antimicrobial guidelines recommending amoxicillin and gentamicin for HAP with ≥2 SIRS criteria or doxycycline for patients with <2 criteria. There was no difference in the time to HAP onset or qSOFA scores between the groups. Empiric therapy constituted doxycycline (95.9%), amoxicillin (2.5%), or amoxicillin plus clarithromycin (1.6%). In all 29 instances of antimicrobial escalation, this represented changing to a regimen with broader Gram-negative coverage.

Logistic regression identified new/increased supplemental oxygen requirements (OR, 10.9; 95% CI, 3.1–51.1; *P* = .0007), prior doxycycline treatment (OR, 8.2; 95% CI, 1.3–73.6; *P* = .03), diabetes mellitus (OR, 7.5; 95% CI, 2.1–33.1; *P* = .004), a neutrophil count >6.2 × 10^9^/L (OR, 3.9; 95% CI, 1.1–16.3; *P* = .04), and a respiratory rate >18/minute (OR, 3.5; 95% CI, 1.2–11.6; *P* = .03) as being associated with treatment failure in patients treated without empiric broad-spectrum Gram-negative coverage.

## Discussion

In this cohort, patients with non-ICU HAP were elderly, had significant comorbidities, and had an overall low severity of illness. Importantly, a large subgroup were treated successfully without broad-spectrum Gram-negative coverage.

Representative case ascertainment is challenging for non-ICU HAP [9]. A strength of this study was patient identification through systematic screening of inpatient CXRs and correlation with clinical data. Relying on the submission of sputum cultures, positive sputum cultures or discharge coding would have failed to identify most patients. The lack of a control group with low-severity HAP treated without antimicrobials is a limitation.

Of all patients, 42.5% had a clinical cure without broad-spectrum Gram-negative coverage, representing 69.7% of patients treated empirically without such coverage. Amongst patients treated without such coverage, treatment failure was associated with diabetes, prior doxycycline treatment during the same admission, a new/increased oxygen requirement, neutrophil count, and respiratory rate. Hypoxia, tachypnoea, and neutrophilia likely relate to disease severity. Diabetes has been associated with increased pharyngeal colonization by GNB and, therefore, could be a risk factor for GNB HAP [10]. Similarly, prior doxycycline treatment could deplete the respiratory tract of doxycycline-susceptible organisms, influencing the etiology of subsequent HAP. Importantly, the time from admission to HAP onset was not associated with treatment failure without broad-spectrum Gram-negative coverage. Often, 5 days of hospital admission is used as a cut-off to define “late-onset” HAP and the risk of hospital-acquired GNB infection, based on ICU studies and expert opinion [1, 2]. Amongst patients in this cohort with a clinical cure without broad-spectrum Gram-negative coverage, the median onset of HAP was 10 days after admission (IQR, 5–21), suggesting the currently recommended 5-day cut-off is not applicable to non-ICU HAP.

In conclusion, using a representative cohort of patients with non-ICU HAP, we report that a large subgroup of patients do not require broad-spectrum Gram-negative coverage. Lower disease severity markers (SIRS, hypoxia, tachypnoea, neutrophilia), an absence of diabetes mellitus, and prior doxycycline treatment (but not time to HAP onset) identify this subgroup.

## Supplementary Data

Supplementary materials are available at *Clinical Infectious Diseases* online. Consisting of data provided by the authors to benefit the reader, the posted materials are not copyedited and are the sole responsibility of the authors, so questions or comments should be addressed to the corresponding author.

ciaa391_suppl_Supplementary_Material

## Notes

***Acknowledgments.*** The authors thank Carol Donaldson for performing the electronic search for chest X-ray reports.

***Financial support.*** C. D. R. is a member of the Medical Research Council SHIELD consortium antimicrobial resistance research consortium and is supported by an Edinburgh Clinical Academic Track (ECAT)/Wellcome Trust PhD Training Fellowship for Clinicians award (award number 214178/Z/18/Z).

***Potential conflicts of interest.*** All other authors report no potential conflicts. All authors have submitted the ICMJE Form for Disclosure of Potential Conflicts of Interest. Conflicts that the editors consider relevant to the content of the manuscript have been disclosed.
