# Supplementary material for: A Subgroup of Patients With Hospital-acquired Pneumonia Do Not Require Broad-spectrum Gram-negative Antimicrobial Coverage
Source: Clin Infect Dis. 2020 Apr 8;71(10):e710–3. doi: 10.1093/cid/ciaa391 (PMC7744989; doi:10.1093/cid/ciaa391)
Supplement: ciaa391_suppl_Supplementary_Material [file ciaa391_suppl_supplementary_material.pdf]

Supplementary Data

A sub-group of patients with hospital-acquired pneumonia do not require broad-spectrum gram-negative antimicrobial coverage

Clark D. Russell[#], Ed Whittaker, Dominic P. Dee, Eilidh Farquhar, Alba Saenz de Villaverde, Morgan H. Evans, Ian F. Laurenson, Claire L. Mackintosh, Muge Cevik

[#]University of Edinburgh Centre for Inflammation Research, Queen's Medical Research Institute, Edinburgh BioQuarter, 47 Little France Crescent, EH16 4TJ, Edinburgh, U.K. Tel: +44 (0)131 2426550. E-mail: clark.russell@ed.ac.uk

Supplementary Table 1: Sputum culture & respiratory PCR results

**Pathogen(s)**	**Number**
**Sputum culture** (n=37)	
No growth/Commensals	18
Pseudomonas aeruginosa[a]	4
Streptococcus pneumoniae & *Haemophilus influenzae*	3
H. influenzae	2
Proteus mirabilis	2
Coliforms	2
Enterococcus faecalis	1
Klebsiella aerogenes	1
K. pneumoniae	1
Escherichia coli & *Serratia marcescens*	1
H. influenzae & *Moraxella catarrhalis*	1
Staphylococcus aureus & *E. coli*	1
**Respiratory PCR throat swab** (n=54)	
Influenza A	5
Parainfluenza type 3	3
Rhinovirus	3
Human metapneumovirus	2
Coronavirus OC43[a]	1
Coronavirus 229E	1
Coronavirus NL63	1
Respiratory syncytial virus	1
Influenza A & respiratory syncytial virus	1
Mycoplasma pneumoniae	0
Chlamydophila pneumoniae	0

[a]one incidence of bacterial/viral co-infection with *P. aeruginosa* & coronavirus OC43.

Supplementary Table 2: Empiric antimicrobials

**Antimicrobial(s)**	**Number (%)**	**Broad-spectrum gram-negative coverage**
Doxycycline	119 (59.5)	**No**
Amoxicillin & gentamicin	28 (14)	Yes
Vancomycin & gentamicin	13 (6.5)	Yes
Piperacillin-tazobactam	11 (5.5)	Yes
Co-trimoxazole	7 (3.5)	Yes
Co-amoxiclav	4 (2)	Yes
Amoxicillin	3 (1.5)	**No**
Meropenem & vancomycin	3 (1.5)	Yes
Amoxicillin & clarithromycin	2 (1)	**No**
Piperacillin-tazobactam & clarithromycin	2 (1)	Yes
Co-amoxiclav & clarithromycin	2 (1)	Yes
Vancomycin & ciprofloxacin	2 (1)	Yes
Ceftazidime & vancomycin	1 (0.5)	Yes
Piperacillin-tazobactam & gentamicin	1 (0.5)	Yes
Piperacillin-tazobactam & vancomycin	1 (0.5)	Yes
Unknown	1 (0.5)	NA
